# miR-424 acts as a tumor radiosensitizer by targeting aprataxin in cervical cancer

**DOI:** 10.18632/oncotarget.12716

**Published:** 2016-10-18

**Authors:** Xia Wang, Qing Li, Hua Jin, Hua Zou, Wei Xia, Nan Dai, Xiao-Yan Dai, Dong Wang, Cheng-Xiong Xu, Yi Qing

**Affiliations:** ^1^ Department of Laboratory Medicine, West China Second University Hospital, Sichuan University, Chengdu, Sichuan 610041, China; ^2^ Laboratory of Obstetric and Gynecologic and Pediatric Diseases and Birth Defects of Ministry of Education, Chengdu, Sichuan 610041, China; ^3^ Cancer Center, Daping Hospital and Research Institute of Surgery, Third Military Medical University, Chongqing 400042, China; ^4^ Department of Thoracic Surgery, Daping Hospital and Research Institute of Surgery, Third Military Medical University, Chongqing 400042, China

**Keywords:** miR-424, aprataxin, radioresistance, cervical cancer

## Abstract

Previous studies have shown that some dysregulated miRNAs are involved in radioresistance of tumor cells. Here, we identified significantly decreased miR-424 expression in radioresistant cervical cancer cells and specimens from cervical cancer patients with radioresistance compared to their radiosensitive parental cells and specimens from radiosensitive patients, respectively. Ectopic expression of miR-424 significantly increased radiation-induced DNA damage, cell apoptosis and G2/M cell cycle arrest in radioresistant cervical cancer cells. Notably, miR-424 agomiR treatment can sensitize radioresistant cervical cancer cells to radiation in a xenograft model. Furthermore, we demonstrated that miR-424 regulated radiosensitivity by directly targeting aprataxin. Taken together, these findings suggest that miR-424 acts as a radiosensitizing miRNA and reveal a new therapeutic strategy for radioresistant cervical cancers.

## INTRODUCTION

Cervical cancer remains one of the leading gynecological malignancies affecting women worldwide [1], and radiotherapy is the most common intervention, as either a primary or adjuvant therapy [2, 3]. However, the 5-year survival rate for advanced-stage cervical cancer patients is 66% [4], suggesting the need to enhance radiosensitivity in cervical cancer. However, the mechanism of cellular radiosensitivity regulation remains unclear in cervical cancer.

Increased DNA repair can result in radioresistance because radiotherapy causes tumor cell death through DNA damage [5]. Recent studies show that radiotherapy can dysregulate some miRNAs, which significantly contributes to the development of radioresistance in cervical cancer [6, 7] by activating DNA repair pathways [8, 9]. miRNAs belong to a class of small noncoding RNA molecules (18–22 nucleotides) that post-transcriptionally modulate gene expression by binding to the 3′-untranslated region (3′UTR) of target gene messenger RNAs (mRNAs) [10, 11]. Because a single miRNA can target hundreds of genes, affecting a large cellular signaling network, single miRNAs play key roles in cancer progression [12]. Thus, investigating the role of each dysregulated miRNA and its corresponding mechanism is very important in cancer [13]. However, our understanding of the potential roles of miRNAs in the radiation treatment response for cervical cancer remains limited.

Dysregulated expression of miR-424 was identified in cervical cancers [14], and a previous study showed that miR-424 was involved in ovarian cancer radiosensitivity [15], suggesting that miR-424 may also be involved in cervical cancer radiosensitivity. However, the effect of miR-424 on radiotherapy sensitivity in cervical cancer is unknown. Here, we describe a functional role of miR-424 as a tumor radiosensitizer in cervical cancer cells. We also identified that aprataxin (APTX) is a target of miR-424 in cervical cancer.

## RESULTS

### miR-424 is negatively related to radioresistance in cervical cancer

**Figure 1:**
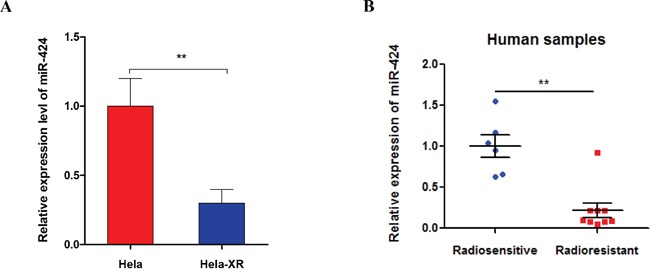
miR-424 expression was decreased in radioresistant Hela cells (Hela-XR) and specimens from cervical cancer patients with radioresistance **A.** The miR-424 expression was measured in Hela-XR cells and their parental Hela cells using RT-qPCR. **B.** The miR-424 expression was measured in specimens of cervical cancer patients with radiosensitive (n=6) and radioresistance (n=9) by RT-qPCR. The data are presented as the mean±SD from three independent experiments.^**^ *p*<0.01.

We first quantified the miR-424 expression level in both radioresistant cervical cancer cells and their parental cells by RT-qPCR. As shown in Figure 1A, the miR-424 expression was significantly suppressed in radioresistant Hela cells (Hela-XR) compared to their parental Hela cells. As in the *in vitro* experiments, clinical data show that miR-424 expression is significantly suppressed in specimens from cervical cancer patients with radiotherapy resistance compared to specimens from radiotherapy sensitive patients (Figure 1B). Taken together, these data suggest that decreased miR-424 might be associated with radioresistance in cervical cancer.

### Ectopic expression of miR-424 enhances the sensitivity of cervical cancer cells to IR treatment *in vitro*

**Figure 2:**
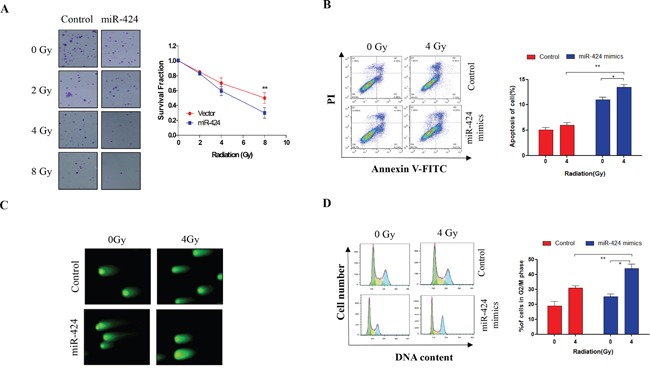
Ectopic expression of miR-424 enhances radiosensitivity of Hela-XR cells Hela-XR cells were transfected with lentiviral miR-424 or vectors and exposed to the indicated doses of irradiation (IR) for 30 minutes after 48 hrs of transfection. Then, **A.** cells were allowed to grow until visible colonies appeared. **B.** After 24 hrs of IR treatment, cells were subjected to apoptosis analysis. **C.** After 4 hrs of IR treatment, cells were subjected to comet assay. **D.** After 24 hrs of IR treatment, cells were subjected to cell cycle analysis. The data are presented as the mean±SD from three independent experiments. ^*^ *p*<0.05 and ^**^ *p*<0.01.

Next, we investigated the impact of miR-424 on the radiosensitivity of cervical cancer cells. Our clonogenic assay show that combined treatment of miR-424 and IR more significantly reduced the survival fraction of Hela-XR cells compared to IR treatment alone (Figure 2A). These results were confirmed using flow cytometric analysis. As shown in Figure 2B, the combination of ectopic miR-424 expression and IR-treatment significantly increases apoptotic Hela-XR cells compared to a single treatment with miR-424 or IR. In addition, a comet assay showed that the combination of miR-424 and IR-treatment more significantly induces DNA damage compared to miR-424 or IR treatment alone in Hela-XR cells (Figure 2C).

Further, we investigated the effects of miR-424 on IR-induced G2/M cell cycle arrest. It has been reported that alterations in cell cycle progression post-IR are associated with the IR-sensitivity of tumor cells [16]. Our data show that 4 Gy IR-treatment does not significantly induce G2/M cell cycle arrest in Hela-XR cells compared to control cells (Figure 2D). However, ectopic miR-424 expression significantly stimulated IR-induced G2/M cell cycle arrest of Hela-XR cells compared to single treatment with miR-424 or IR (Figure 2D). These findings suggest that miR-424 can enhance the radiosensitivity of radioresistant cervical cancer cells by promoting IR-induced DNA damage, apoptosis and G2/M cell cycle arrest.

### miR-424 enhances the radiosensitivity of cervical cancer in xenograft models

**Figure 3:**
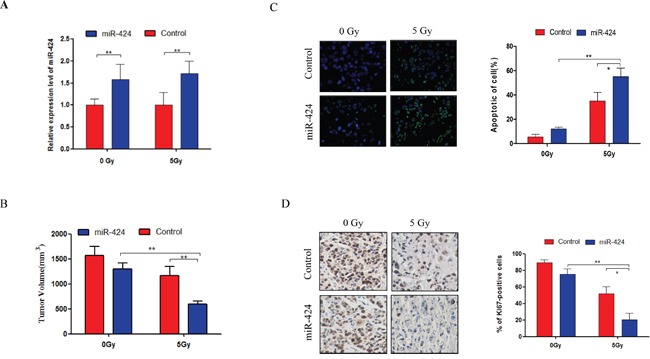
Combined treatment of miR-424 and IR significantly suppressed tumor growth *in vivo* **A.** The miR-424 expression was measured from xenograft tumors by RT-qPCR. **B.** Xenograft tumor volume. One week after Hela-XR cell injection, mice were treated with miR-424 agomir or control agomir every 3 days for 7 times. Then, mice were exposed 3 times to 5 Gy IR or not every 3 days. The tumor volume was measured every 3 days and mice were sacrificed after 37 days of cell injection. **C.** TUNEL assay and **D.** Ki67 immunostaining were performed in xenograft tumor tissues. The data are presented as the mean±SD.^*^ *p*<0.05 and ^**^ *p*<0.01.

To further identify the therapeutic potential of miR-424 as a tumor radiosensitizer *in vivo*, we performed xenograft studies. We used Hela-XR cells in a xenograft model and delivered miR-424 agomir by direct intratumoral injection (Figure 3A). Overexpression of miR-424 slightly suppressed tumor growth compared to control, but there was no significant difference. However, the combination of miR-424 and IR-treatment significantly suppressed tumor growth compared to a single treatment with miR-424 or IR (Figure 3B). In addition, TUNEL and IHC assay data showed that the combination of miR-424 and IR-treatment more significantly increased apoptosis (Figure 3C) and inhibited cell proliferation compared to a single treatment with miR-424 or IR-treatment (Figure 3D). Collectively, these data suggest that miR-424 acts as a radiosensitizer *in vivo*.

### miR-424 directly targets APTX in cervical cancer

**Figure 4:**
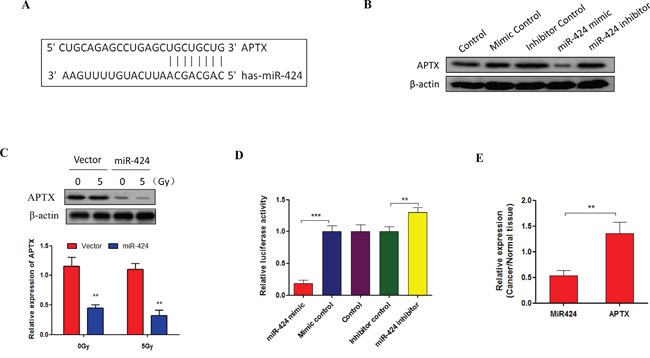
APTX is a target of miR-424 in cervical cancer **A.** Sequence alignment of miR-424 with the 3′UTR of the APTX gene. **B.** Hela cells were transfected with indicated nucleotides. After 48 hrs of transfection, APTX expression was measured by Western blot. **C.** APTX expression was measured by RT-qPCR and Western blot in xenograft tumors from miR-424-overexpressing Hela-XR cells and vector control Hela cells. **D.** Hela cells were cotransfected with APTX 3′UTR luciferase reporter construct and the indicated nucleotides. After 48 hrs of transfection, the luciferase intensity was assessed. The data are presented as the mean±SD from three independent experiments. ^**^ *p*<0.01. **E.** The relative expression of APTX and miR-424 was measured in specimens of cervical cancer patients by RT-qPCR (n=10).

To investigate the mechanism of miR-424 in regulating cervical cancer cell radiosensitivity, we used algorithms that predict mRNA targets of miRNAs that have been identified as candidates for miR-424, and we choose APTX as a putative target gene for miR-424 (Figure 4A). APTX stimulates DNA repair and protects cells against genotoxic stress in cancer cell [17, 18]. Our data show that APTX expression was significantly suppressed by ectopic expression of miR-424 and that the expression was increased by miR-424 inhibition in Hela cells (Figure 4B). Consistent with this, an *in vivo* experiment also showed that APTX expression was significantly suppressed in xenograft tumors by miR-424 overexpression (Figure 4C), suggesting that APTX was negatively regulated by miR-424. Furthermore, we conducted a luciferase reporter assay to demonstrate the direct binding of the miR-424 and APTX 3′UTR region. The 3′UTR of APTX, which was harboring the complementary sequence for the miR-424 seed sequence, was cloned into a luciferase reporter plasmid. Transient cotransfection of the APTX-3′UTR construct with miR-424 into Hela cells led to a significant decrease in firefly luciferase activity compared to the control group. In contrast, cotransfection of the APTX-3′UTR construct with miR-424 inhibitor into Hela cells led to a significant increases in firefly luciferase activity compared to the control group (Figure 4D). In addition, we identified a negative correlation between miR-424 and APTX expression in specimens from patients with cervical cancer (Figure 4E). Taken together, these data suggest that APTX is target gene of miR-424 in cervical cancer.

### Silencing of APTX enhances cervical cancer radiosensitivity

**Figure 5:**
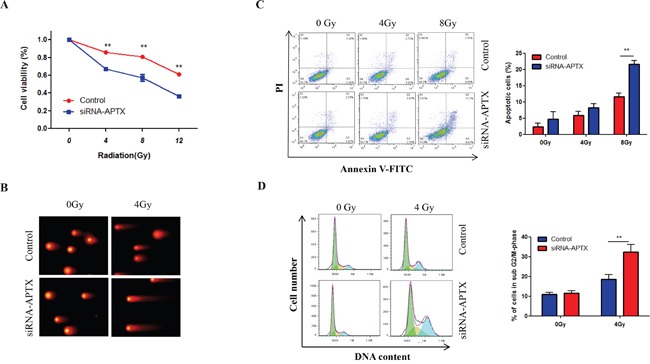
Silencing of APTX enhanced the radiosensitivity of Hela-XR cells Hela-XR cells were transfected with APTX siRNA or control nucleotide and they were exposed to the indicated doses of IR after 48 hrs of transfection. **A.** After 24 hrs of IR, cell viability was measured with a CCK-8 kit. **B.** After 24 hrs of irradiation, apoptotic cells were detected by flow cytometric analysis. **C.** After 4 hrs of IR, a comet assay was performed. **D.** After 24 hrs of IR, cells were subjected to cell cycle analysis. The data are presented as the mean±SD from three independent experiments. ^**^ *p*<0.01.

The above results indicate that miR-424 enhances the radiosensitivity of cervical cancer and that APTX is a target of miR-424, suggesting that decreased APTX can enhance radiosensitivity in cervical cancer. However, there is no direct evidence for the role of APTX in cervical cancer radiosensitivity. Thus, we investigated the effects of APTX on cervical cancer radiosensitivity. As shown in Figure 5A, silencing of APTX significantly suppressed cell viability after IR-treatment in Hela-XR cells (Figure 5A). In addition, that combination of APTX-silencing and IR-treatment more significantly induced DNA damage (Figure 5B) and apoptosis compared to IR alone treatment in Hela-XR cells (Figure 5C). Furthermore, cell cycle analysis showed that the combination of IR and APTX-silencing more significantly induced G2/M cell cycle arrest in Hela-XR cells compared to IR treatment alone (Figure 5D). These findings suggest that inhibiting APTX can enhance cervical cancer radiosensitivity.

### miR-424 inhibits radioresistance through targeting APTX in cervical cancer cells

**Figure 6:**
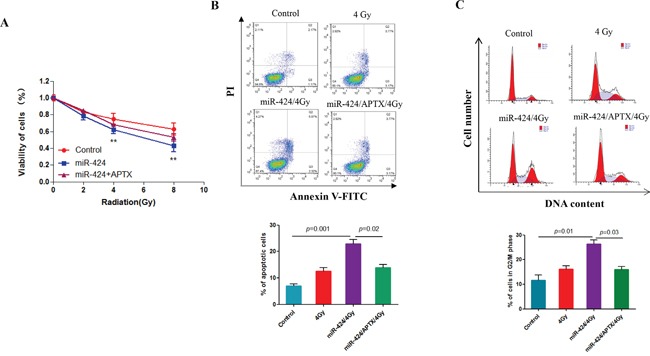
miR-424 enhances radiosensitivity through APTX in cervical cancer cells Hela-XR cells were transfected with the indicated plasmid and/or nucleotides. After 48 hrs of transfection, cells were exposed to the indicated doses of IR. After 24 hrs of IR, cells were subjected to analysis of **A.** cell viability, **B.** apoptosis, and **C.** cell cycle. The data are presented as the mean±SD from three independent experiments.

Here, we also investigated whether APTX directly contributes to the role of miR-424 for radioresistant cervical cancer cells. As shown in Figures 6A and 6B, ectopic expression of APTX can block cell viability inhibition and apoptosis that are induced by miR-424 overexpression after IR-treatment in Hela-XR cells. Additionally, APTX overexpression blocked miR-424, and IR-treatment induced cell cycle arrest (Figure 6C), suggesting that APTX is the major contributor to the effects of miR-424 on cervical cancer cell radiosensitivity.

## DISCUSSION

Over 60% cases of patients with cervical cancer receive radiotherapy. Thus, it is very important to enhance the radiotherapy effect [19]. Here, we observed, for the first time, that the miR-424 expression was remarkably decreased in specimens from cervical cancer patients with radioresistance compared to specimens from radiosensitive patients. Additionally, we demonstrated the crucial role of miR-424 in enhancing cervical cancer radiosensitivity. Notably, our investigation further demonstrated that ectopic expression of miR-424 can significantly enhance the radiosensitivity of radioresistant cervical cancer cells *in vitro* and *in vivo*, which suggests that the ectopic expression of miR-424 may be a novel strategy for enhancing radiosensitivity in cervical cancer patients.

In this study, we also clarified the mechanism of miR-424 in regulating radiosensitivity in cervical cancer. Here, we identified that miR-424 can dramatically enhance the radiosensitivity of radioresistant cervical cancer cells through stimulating IR-induced DNA damage, apoptosis and G2/M cell cycle arrest. Furthermore, we identified that miR-424 exhibits its biological function through directly inhibiting the expression of APTX in cervical cancer cells. APTX is a DNA repair-related protein that can stimulate the repair of DNA strand breaks caused by various DNA damaging agents [20]. Studies show that increased APTX expression was closely associated with anticancer drug resistance in cervical carcinoma cells [21]. Consistent with this report, our data show that inhibiting APTX can stimulate IR-induced DNA damage, apoptosis and G2/M cell cycle arrest in cervical cancer cells. In addition, enhanced radiosensitivity by miR-424 was abolished by ectopic expression of APTX in cervical cancer cells. These findings clearly demonstrate that APTX is a key downstream effector in mediating the effects of miR-424 on radiosensitivity and that APTX is also a novel therapeutic target for enhancing radiotherapy effects in cervical cancer patients.

In summary, this study identified novel roles of miR-424 in regulating cervical cancer cell radiosensitivity. miR-424 sensitizes the radioresistant cervical cancer cells to radiotherapy by inhibiting APTX expression. Our findings help establish new strategies for improving the therapeutic effects of treatments for cervical cancer patients with radiation resistance.

## MATERIALS AND METHODS

### Cell culture and transfection

Hela and Hela X ray resistance (Hela-XR) cells were maintained in Dulbecco's modified Eagle's medium with 10% fetal bovine serum (FBS; HyClone, Logan, UT) at 37 °C in an atmosphere with 95% air and 5% CO_2_. The Hela-XR cell line was previously developed by our laboratory [22]. Cells were transfected with indicated nucleotides or plasmid using Lipofectamine 2000 (Invitrogen, CA, USA) according to manufacturer's instructions.

### Cell viability and clonogenic assay

Cells were plated in a 96-well plate at a density 4,000 cells/well and transfected with indicated nucleotides or plasmid. After 48 hrs of transfection, cells were exposed to indicated doses of irradiation (IR). Twenty-four hours after IR treatment, cell viability was determined using a CCK-8 kit (Dojindo Laboratories, Kumamoto, Japan) according to the manufacturer's protocol.

For the clonogenic assay, cells were seeded in a 60-mm^2^ dish at a density of 1,0000 cells/plate and transfected with lentiviral miR-424 or empty lentiviral vector. After 48 hrs of transfection, cells were exposed to indicated doses of IR; then, cells were allowed to grow until visible colonies appeared. The colonies were stained with Giemsa (Abcam, Cambridge, MA, USA) and counted.

### Apoptosis and cell cycle analysis

Cells were transfected with indicated nucleotides, and plasmids and cells were exposed to indicated doses of IR after 48 hrs of transfection. Twenty-four hours after irradiation, cells were subjected to apoptosis and cell cycle analysis. Apoptotic cells were determined by flow cytometric analysis using Annexin V-FITC kit (Calbiochem, Shanghai, China) according to the manufacturer's instructions. For the cell cycle analysis, IR-treated cells were harvested by trypsinization, washed twice using cold PBS and fixed in 70% ethanol overnight at -20 °C. Then, cells were treated with DNA staining solution, and cell cycle analysis was performed with FACS flow cytometry.

### Comet analysis

Cells were transfected with indicated oligonucleotides and plasmid. After 48 hrs of transfection, cells were exposed to indicated doses of IR. Four hours after IR, cells were harvested and diluted into 4 × 10^4^/ml. Samples were placed on a rubber sheet after the cells were mixed with 0.5% low melting point agarose; in the horizontal electrophoresis tank, a fresh liquid preparation of cold electrophoresis buffer was poured. The DNA chain solutions were prepared in 30 minutes; then, electrophoresis was performed for 25 minutes (20 V, 300 mA); the glasses were washed with neutral buffer 3 times (5 min each) after electrophoresis. The glasses were dried, stained with 10-15 μl of EB (Ethidium bromide) (20 ug/ml) liquid, observed by fluorescence microscopy, and then the comets were analyzed with Komet 5.5 software.

### Luciferase reporter assay

The aprataxin (APTX) 3′UTR, which contained target sequences complementary to the miR-424 seed sequence, was cloned downstream of the firefly luciferase gene in the pMIR-REPORT luciferase vector (Ambion, Cambridge, MA, USA). The APTX reporter constructs andindicated oligonucleotides were cotransfected with the phRG-TK Renilla luciferase internal control plasmid (Promega, Madison, WI, USA) into Hela cells. Cell extracts were prepared 48 hrs after transfection, and the luciferase activity was measured using the Dual-Luciferase Reporter Assay System (Promega) according to manufacturer's instructions.

### Real-time quantitative reverse transcriptase polymerase chain reaction analysis

Total RNA was isolated form cell and fresh tissue samples using the TRIzol reagent (Invitrogen) according to the manufacturer's protocol. Mature miR-424 and the RNU6 endogenous control were analyzed using the TaqMan microRNA Assay Kit (Applied Biosystems, Foster City, CA, USA). The relative expression of miR-424 was normalized against RNU6 expression using the 2^−ΔCt^ method. To analyze other genes expression, RT and PCR were performed with a high-capacity cDNA reverse transcription kit and QuantiTect SYBR Green PCR kit (Qiagen), respectively. The primer sequences for genes were defined as follows: APTX forward, 5′-CAGGGAACACCTTGAACTCC-3′ and reverse, 5′-ATACTCGGAATGGCGTGGTA -3′ and GAPDH forward, 5′-GCAGGGGGGAGCCAAAAGGGT-3′ and reverse, and 5′-TGGGTGGCAGTGATGGCATGG-3′.

### Western blot and immunohistochemistry (IHC)

Western blot [23] and IHC [24] were performed as previously described. anti-APTX antibody was purchased from Gene Tex (Irvin, CA, USA); anti-β-actin antibody, anti-Ki67 antibody and secondary antibodies conjugated to horseradish peroxidase (HRP) were obtained from Abcam.

### Human specimens and animal experiments

This study was approved by the Ethics Committee of the Third Military Medical University, and clinical samples were obtained from Daping Hospital and Research Institute of Surgery, The Third Military Medical University. Samples were collected by biopsy from patients before and after radiotherapy. First, samples were prepared to measure gene expression. Then, the second samples (after radiotherapy) underwent evaluation of the radiotherapy effect. The radiotherapy effect was evaluated according to methods by Kitahara et al. [25], and samples were divided into radiosensitive and radioresistant groups based on the evaluation results for radiotherapy.

For the tumor xenograft assay, 5-weeks-old female BALB/c-nude mice were purchased from Shanghai Laboratory Animal Center (Shanghai, China) and housed under specific pathogen-free conditions. All animal experiments were approved by the Animal Care Committee of the Third Military Medical University. To establish a xenograft model, we subcutaneously injected 1.5 × 10^7^ Hela-XR cells (each side) in 100 μl of phosphate-buffered saline (PBS) into the bilateral armpits of the nude mice. The tumor size was monitored twice a week with calipers. One week after tumor cell inoculation, the mice were divided into two groups (Control and radiotherapy groups; n= 6/group). For agomir treatment, miR-424 agomir (4 ng/mm^3^tumor) and miR-NC agomir were directly, intratumorally injected into the right and left sides of the mice, respectively. Mice were treated 7 times with agomir every 3 days. At day 28, the animals were irradiated with 5 Gy or not every three days for three times. At day 37, all mice were sacrificed, and the tumors were excised.

### Statistical analysis

*p*<0.05 was considered statistically significant. All assays were performed at least three times independently. Values are presented as the mean with standard deviation (SD). ANOVA was used to evaluate the comparisons of multiple groups by one-way analysis.
